# Magnitude and associated factors of virological failure among children on ART in Bahir Dar Town public health facilities, Northwest Ethiopia: a facility based cross-sectional study

**DOI:** 10.1186/s13052-021-01030-7

**Published:** 2021-04-06

**Authors:** Belete Gelaw, Getasew Mulatu, Getasew Tesfa, Chalie Marew, Bogale Chekole, Animut Alebel

**Affiliations:** 1 School of Nursing, College of Medicine and Health Sciences, Wolaita Sodo University, Sodo, Ethiopia; 2 College of Medicine and Health Sciences, Bahir Dar University, Bahir Dar, Ethiopia; 3 College of Medicine and Health Sciences, Debre Tabor University, Debre Tabor, Ethiopia; 4 College of Medicine and Health Sciences, Wolkite University, Wolkite, Ethiopia; 5 College of Health Science, Debre Markos University, Debre Markos, Ethiopia; 6 School of Public Health, Faculty of Health, University of Technology Sydney, Ultimo, NSW Australia

**Keywords:** Antiretroviral therapy, Bahir Dar, Children, HIV, Virological failure

## Abstract

**Background:**

Despite the rapid scale-up of antiretroviral therapy, virologic failure has become global public health concern and challenge, especially in developing countries. Viral load monitoring is an important approach to identify treatment failure and develop public health interventions in children receiving antiretroviral therapy. Thus, this study aims to assess the magnitude and associated factors of virological failure among children on antiretroviral therapy.

**Methods:**

A facility-based cross-sectional study was conducted among 399 HIV-positive children on antiretroviral therapy from 2016 to 2019 in Bahir Dar Town public health facilities. Data were extracted from children’s charts using a standardized data extraction tool, adapted from ART intake and follow-up forms. Data were entered using Epi-Data Version 3.1, and analyzed using SPSS Version 25. Bivariable and multivariable binary logistic regression models were done to identify factors associated with virological failure. Variables with *p*-values < 0.25 were fitted into the multivariable analysis. Finally, variables with *p*-values <0.05 were considered as statistically significant factors.

**Results:**

The period prevalence of virological failure was found to be 14.8% (95% CI: 11.5–19.3%). Opportunistic infections (AOR = 2.19, CI: 1.13–4.25), history of treatment interruption and restart (AOR = 2.21, CI: 1.09–4.54), younger age (AOR = 2.42, CI: 1.02–5.74), poor/fair ART adherence (AOR = 2.19, CI: 1.05–4.57), and advanced baseline WHO clinical staging (AOR = 2.32, CI: 1.14–4.74) were found to be factors significantly associated with virological failure.

**Conclusion:**

The magnitude of virological failure among HIV-infected children remained high. Children with poor/fair ART adherence, history of treatment interruption, advanced baseline WHO clinical staging, younger age, and opportunistic infections were significantly associated with virologic failure. Thus, special attention should be given to children who had poor/fair ART adherence and presenting with opportunistic infections.

## Background

Globally, human immunodeficiency virus (HIV) is a major public health problem and associated with a range of short-and long-term consequences. Approximately 1.8 million children (age < 15 years) were living with HIV globally in 2018 [1]. Sub-Saharan Africa (SSA) suffers from the global burden of HIV- infection, with nearly 70% of the world’s HIV/AIDS-infections and deaths occurs in this region [2]. Based on Ethiopian public health institution HIV related estimates and projections about 704,454 populations were living with HIV in 2020. Of these 41,265 were children. Beside that 20,130 were newly infected and 7684 were died with HIV related illness [3].

The goal of antiretroviral therapy (ART) is to suppress viral replication and reduce HIV associated mortality among infected children. Thus, monitoring people on ART is important to ensure treatment success, identify adherence problems, and determine whether ART regimens should be switched in case of treatment failure (TF). Treatment failure can be assessed in three ways: clinically, immunologically, and virologically, which offers early and accurate indication of treatment failure [4].

The World Health Organization (WHO) recommends routine annual viral load monitoring for all patients on ART, as the most accurate and preferred method to measure treatment response [4, 5]. In 2014, the Joint United Nations Program on HIV/AIDS (UNAIDS) and partners launched the three 90–90–90 targets, having 90% of patients on ART with VLS is the third “90” targets. Adherence counseling, early detection of TF, and appropriate switching to second-line therapy are key strengths of a viral load monitoring [4, 6]. Likewise, the Ethiopian government launched different strategies, programs, and policies that provide aspiring and far-reaching goals on prevention, treatment, health coverage, and access to affordable medicine for HIV/AIDS patients like Sustainable Development Goals (SDGs) [7]. Ethiopia has adopted the UNAIDS 90–90-90 HIV treatment target and has developed HIV/AIDS prevention and strategic treatment plan, which has been implemented since 2015 [5]. Ending the AIDS epidemic by 2030 is the aim of this strategic plan in line with the three 90’s targets.

In Ethiopia, few studies have been conducted on TF by considering previous guidelines [8]. However, some critical factors such as HIV status disclosure, level of Hgb, history of ART interruption, CD4 cell counts, and caregiver’s HIV status, which contribute to VF occurrence, were not included. Furthermore, the previous studies were done at the referral hospitals with diverse health workers to manage cases [8, 9]. Despite hospitals and health centers are using the same ART guidelines, this study included health centers as they cover most health facilities that have been giving pediatric ART services [5].

Though, different studies have been conducted on survival, ART adherence, and other aspects of HIV**-** infected children, the magnitude and associated factors of VF among children on ART has not yet been well investigated in Ethiopia. Current and up-to-date information regarding virological failure (VF) in HIV-positive children using routine viral load testing to measure treatment failure is essential for policy makers to take appropriate actions. Therefore, the findings of this study will highlight the prevalence and associated factors of VF with implications to improve health workers’ interventions, to ensure treatment cost-effectiveness, and to accelerate the reduction of HIV related morbidity and mortality of children.

## Methods

### Study settings, design, and period

A health facility-based cross-sectional study was carried out from February to March 2020 among HIV-infected children on ART between September 2016 and December 2019. In Ethiopia, chronic HIV/AIDS care and treatment services are provided at hospital and health center settings throughout the country. This study was conducted in Bahir Dar town public health facilities, which is 565 km away from Addis Ababa. The study was conducted in four selected public health facilities (i.e. Bahir Dar Health Center, Abay Health Center, Han Health Center, and Felege Hiwot Comprehensive Specialized Hospital).

### Study participants, sample size, and sampling technique

All HIV-infected children (aged < 15 years) taking ART in Bahir Dar Town public health facility were the target population. All HIV-infected children who took ART at least for 9 months with documented viral load test results were included. Conversely, children with incomplete medical records (i.e. age of child at ART initiation, baseline ART regimen, current CD4 cell counts and unknown outcome status) were excluded.

The minimum required sample size was determined using a single population proportion formula. To compute the sample size, the following statistical assumptions were considered: - prevalence of VF =50% because there was no published study done on this topic in Ethiopia, margin of error = 5% and the value of Zα/2 = 1.96, which is the corresponding Z score of 95% confidence interval (CI).

$$ \mathrm{n}=\frac{{\left(\mathrm{Za}/2\right)}^2\mathrm{p}\left(1-\mathrm{p}\right)}{{\left(\mathrm{d}\right)}^2}=\frac{(1.96)^2\ 0.5\left(1-0.5\right)}{(0.05)^2}=384.16\sim 385 $$

Where, n = the required sample size, Zα/2 = Standard normal variation for type 1 error, p = prevalence (0.5) & d = Margin of sampling error tolerated (0.05).

The calculated sample size was 385. By considering a 10% contingency rate for incomplete charts, the final minimum required sample size of this study was 424 medical records.

**Fig. 1:**
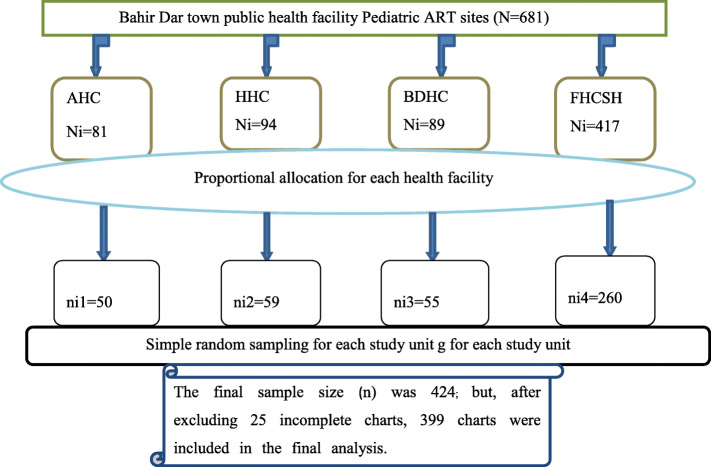
Schematic presentation of sampling procedure to assess the magnitude and associated factors of virological failure among children on ART in Bahir Dar Town public health facility ART clinics (2016–2019), North-west Ethiopia, 2020. Footnote: proportion was calculated using the formula $\mathrm{ni}=\mathrm{Ni}\ \left(\frac{\mathrm{n}}{\mathrm{N}}\right)$ as follows: our calculated sample size (**424**) multiplied by the total number of HIV-infected children on ART at each health facility between 2016 and 2019 (**Ni**), then divided by the total number of HIV-infected children (**N**) in the selected public health facilities. For example, for AHC, it was calculated as: 81*424/681 = 50

This study was conducted in three randomly selected health centers, and one purposively (because it has high patient flow and case-team composite) selected comprehensive specialized hospital. First, a sampling frame was prepared based on the patient’s medical registration number (MRN) from each health facility’s recorded documents. The total sample sizes were then allocated for each healthy facility proportionally to the number of population size. Finally, medical charts of HIV-infected children taking ART at the selected public health facilities were sorted and selected using a simple random sampling technique (Fig. 1**).**

### Data extraction procedure

Available information from patient’s chart was extracted using structured checklist, prepared in English. The data extraction checklist was adapted from the Ethiopian Federal Ministry of Health ART clinic intake and follow-up forms and included socio-demographic, clinical, laboratory, and treatment-related characteristics. Data were extracted by trained health professionals through document review. Five ART trained nurses were recruited as data collector. Orientation about the objectives of the study, contents of the tool, and data extraction procedures was given for data collectors and supervisors for 1 day. The assigned supervisors and principal investigator closely monitored the whole data collection process. Besides, the consistency between collected data and medical records was checked using randomly selected reviews of previously extracted charts.

### Measurements

#### Virologic failure

Was diagnosed when the viral load above or equal to 1000 copies/ml under ART based on two consecutive VL results 3 months apart, with adherence support following the first viral load test after at least 6 months of ART [5].

#### Adherence

Was defined as “good”, “fair” or “poor” if the patient took ≥95% (missing one from 30 doses or two out of 60 doses), 85–94% (missing 2–4 doses out of 30 doses or 4–9 from 60 doses) or <85% (missing ≥5 doses from 30 doses or > 10 from 60 doses), respectively of monthly doses [4].

#### Malnutrition

Was diagnosed when HIV-positive child has one of the following forms of malnutrition: Height/Age < − 2, or Weight/Age < − 2, or Weight/Height < − 2 standard deviation based on the WHO curve [10].

#### Treatment interruption

Was defined as a treatment interruption for at least 1 week during the previous 6 months.

### Data management and statistical analysis

Data were entered into Epi Data Version 3.1 and exported to Statistics Package for Social Science (SPSS) Version 25 for further analysis. Tables were used to present descriptive results. Additionally**,** frequencies, percentages, proportions, and summary statistics (mean, median) were used to summarize the study population characteristics. Variables with *p*-values < 0.25 in the bivariable analysis were entered into the multivariable analysis to control the effects of confounders. Goodness of fit of the model was checked using Hosemer-Lemeshow goodness of fit test. In the multivariable analysis, variables with *p*-values less than 0.05 were considered as statistically significant factors. Lastly, odds ratios with their correspondence 95% CIs were used to assess the strength and the direction of association.

## Results

### Socio-demographic characteristics of the participants

After reviewing 424 HIV-infected children’s charts, 399 charts were included and 25 charts were excluded (six charts missed, and 19 charts had incomplete data) in the final analysis. The medical record retrieval rate was 94.1%. More than half (62.4%) of the study participants were recruited from Felege Hiwot Comprehensive Specialized Hospital. The mean age of children at ART initiation was 6.66 years (SD: ±3.49). Majority (82%) of the children were from Bahir Dar Town. Nearly half of the respondents (48.6%) were females (Table 1).

**Table 1:** Socio-demographic characteristics of the caregivers and study participants in Bahir Dar Town, North western Ethiopia, 2020 (*n* = 399)

Variables	Category	Frequency(n)	Percentage (%)
Age	0–5 years	157	39.3
	6–9 years	147	36.8
	10–15 years	95	23.8
Sex	Female	194	48.6
	Male	205	51.4
Place of residence	Bahir Dar	327	82.0
	Outside of Bahir Dar	72	18.0
Type of health facility	Hospital	249	62.4
	Health centers	150	37.6
Age of caregivers	20–40 years	320	80.2
	> 40 years	79	19.8
Caregivers occupation (*n* = 380)	Government employed	85	21.3
	Housewife	129	32.3
	Merchant	96	24.1
	Daily laborer	37	9.3
	Others	33	8.3
Parent status	Both alive	256	64.2
	Mother died but, father alive	49	12.3
	Father died but, mother alive	53	13.3
	Both died	22	5.5
	Not recorded	19	4.8
Relationship	Parent	364	91.2
	Grandparent	18	4.5
	Others	17	4.3
Caregiver’s HIV Status	Negative	63	15.8
	Positive	303	75.9
	Not recorded	33	8.3
Child’s HIV status disclosure	Disclosed	281	70.4
	Not disclosed	118	29.6

### Clinical, laboratory, and ART information

About 15% of the study participants were taking ART for less than 25 months, and the mean duration on ART was 57.76 months (SD: ±25.81). Majority (83%) of the participants had good level of ART adherence. Regarding nutritional status, only 7% of the children were malnourished. Of these 25% were severely malnourished. More than half (61.7%) of the children had CD4 counts ≥350 cells/mm3, with a median of 453 (IQR: 288–735) cells/mm3. More than half (52.1%) of the children were taking nevirapine-based ART drugs. Additionally, about 14.5% of the children were anemic (Hgb < 10 mg/dl). Nearly, 16.5% of the participants restarted ART after treatment interruption. Furthermore, almost 81% of the participants had history of regimen change due to different reasons. Lastly, most (82.5%) of the participants were classified as WHO clinical stage I/II at ART initiation (Table 2).

**Table 2:** Clinical and treatment-related characteristics of the study subjects at ART initiation and during follow up in Bahir Dar Town public health facilities, Northwestern Ethiopia, 2020(*n* = 399)

Variables	Category	Frequency(n)	Percentage (%)
History of malnutrition	No	371	93.0
	Yes	28	7.0
Severity of malnutrition	Mild	9	32.1
	Moderate	12	42.9
	Sever	7	25.0
Baseline CD4 counts (*n* = 383)	< 350cells/mm3	137	34.3
	≥ 350cells/mm3	246	61.7
Baseline hemoglobin level(*n* = 380)	< 10 mg/dl	58	14.5
	≥ 10 mg/dl	321	80.5
Duration on ART	< 25 months	61	15.3
	≥ 25 months	338	84.7
Baseline WHO clinical staging	Stage I/II	329	82.5
	Stage III/IV	70	17.5
WHO clinical staging in last follow up	Stage I/II	378	94.7
	Stage III/IV	21	5.3
Presence of opportunistic infections	No	272	68.2
	Yes	127	31.8
History of IPT	No	257	64.4
	Yes	142	35.6
History of CPT	No	196	49.1
	Yes	203	50.9
ART regimen given at first	Nvp based	208	52.1
	Efv based	162	40.6
	Others	29	7.3
Regimen change	No	77	19.3
	Yes	322	80.7
History of ART interruption and restart	No	333	83.5
	Yes	66	16.5
Baseline developmental status for age <5 years (*n* = 157)	Appropriate	138	87.9
	Regression	4	2.5
	Delay	15	9.6
Baseline functional status for age ≥ 5 years(*n* = 242)	Working	189	78.1
	Ambulatory	48	19.8
	Bed Ridden	5	2.1

### Magnitude of virological failure

A total of 59 HIV-infected children had a virological failure, verified through record review. The overall magnitude of virologic failure among HIV-infected children was found to be 14.8% (95% CI: 11.5, 19.3%). Most (90%) of the VF occurred after 25 months of ART initiation.

### Factors associated with virological failure

In the multivariable logistic regression analysis, poor ART drug adherence level, treatment restart after the interruption, younger age at ART initiation, history of opportunistic infections, and advanced baseline WHO clinical staging (stage III and IV) were factors significantly associated with the occurrence of virologic failure. Accordingly, the odds of virological failure among children classified as WHO clinical stage III and IV was 2.3 times more likely (AOR = 2.3, 95% CI: 1.1–4.7) compared to their WHO clinical stage I and II counterparts. Moreover, the odds of virological failure among children who had opportunistic infections during follow-up was two times higher as compared to those who did not have OIs (AOR = 2.2,95% CI: 1.1–4.3). Similarly, the odds of virological failure among younger age (age ≤ 5 years) was 2.4 times higher odds (AOR = 2.4, 95% CI: 1.0–5.7) compared to older age children (age 10–15 years). The odds of virological failure was 2.2 times higher (AOR = 2.2, 95% CI: 1.1–4.5) among children who had history of treatment interruption compared with those who did not have history of treatment interruption. Finally, the odds of virological failure among participants who had poor/fair ART adherence level was 2.2 times higher (AOR = 2.2, 95% CI: 1.1–4.6) compared to those who had good ART adherence level (Table 3).

**Table 3:** Bivariable and multivariable logistic regression analysis of factors associated with virological failure among HIV positive children in Bahir Dar Town public health facility ART clinics, Northwestern Ethiopia, 2020 (*n* = 399)

Factors	Virological failure,n (%)		COR(95% CI)	AOR(95% CI)
	Yes	No		
Age				
0–5 years	34 (21.7)	123 (78.3)	2.35 (1.10–5.01)	2.42 (1.02–5.74)**
6–9 years	15 (10.2)	132 (89.8)	0.97 (0.42–2.25)	1.04 (0.42–2.58)
10–15 years	10 (10.5)	85 (89.5)	1	1
Sex				
Male	35 (17.1)	170 (82.9)	1.46 (0.83–2.56)	1.27 (0.68–2.36)
Female	24 (12.4)	170 (87.6)	1	1
Place of residence				
Outside of Bahir Dar	14 (19.4)	58 (80.6)	1.51 (0.78–2.94)	1.80 (0.84–3.86)
Bahir Dar	45 (13.8)	282 (86.2)	1	1
Type of health facility				
Health-center	27 (18)	123 (82)	1.49 (0.85–2.60)	1.69 (0.89–3.22)
Hospital	32 (12.9)	217 (87.1)	1	1
Child’s HIV status disclosure				
Not disclosed	23 (19.5)	95 (80.5)	1.65 (0.93–2.93)	0.85 (0.42–1.72)
Disclosed	36 (12.8)	245 (87.2)	1	1
Baseline WHO clinical Staging				
Stage III/IV	21 (30)	49 (70)	3.28 (1.78–6.06)	2.32 (1.14–4.74)**
Stage I/II	38 (11.6)	291 (88.4)	1	1
Opportunistic infection				
Yes	33 (26)	94 (74)	3.32 (1.89–5.85)	2.19 (1.13–4.25)**
No	26 (9.6)	246 (90.4)	1	1
WHO clinical Staging (last visit)				
Stage III/IV	7 (33.3)	14 (66.7)	3.14 (1.21–8.13)	1.36 (0.44–4.16)
Stage I/II	52 (13.8)	326 (86.2)	1	1
Regimen change/substitute				
Yes	51 (15.8)	271 (84.2)	1.62 (0.74–3.58)	1.53 (0.63–3.72)
No	8 (10.4)	69 (89.6)	1	1
Interruption and restart				
Yes	18 (27.3)	48 (72.7)	2.67 (1.42–5.03)	2.21 (1.09–4.54)**
No	41 (12.3)	292 (87.7)	1	1
ART adherence				
Fair/Poor	20 (29.4)	48 (70.6)	3.12 (1.68–5.80)	2.19 (1.05–4.57)**
Good	39 (11.8)	292 (88.2)	1	1
Duration on ART				
≥ 25 months	53 (15.7)	285 (84.3)	1.71 (0.70–4.16)	2.17 (0.81–5.86)
< 25 months	6 (9.8)	55 (90.2)	1	1

## Discussion

This facility-based cross-sectional study found that the magnitude of virological failure among HIV-infected children was 14.8% (95% CI: 11.5–19.3%). This finding is in line with the magnitude of virological failure reported from Ghana (15.7%%) [11], South Africa (19.3%) [12], and Malawi (16%) [13]. Conversely, this finding is lower than studies conducted in Cameroon (30.6%) [14], Kenya (43.1%) [15], Tanzania (25.4%) [16], and Malawi (66%) [17]. The possible sources of variation in the prevalence rate of virological failure might be due to the differences in sample size, study period, the variability in virological failure measurements, and the difference in diagnostic capacity. As an example, a Tanzanian study included only 206 participants. Regarding variability in virological failure measurements, a studies done in Malawi and Cameroon used a single plasma viral load above 1000 copies/ml, and 200copies/ml to diagnose virological failure respectively [14, 17], whereas this study used two consecutive plasma viral load 1000 copies/ml and above. The other possible justification for the discrepancy could be due to the difference in study period as there were changes in treatment guidelines over time. An additional possible explanation might be the difference in diagnostic capacity between study settings or different methods used to retrieve and analyze it.

We found that different factors were significantly associated with the occurrence of virological failure. In this regard, younger ages (age ≤ 5 years) were more prone to develop virologic failure. This finding is in agreement with other previous studies done in Swaziland [18], African and Asian children [19], Tanzania [20], and Zimbabwe [21]. This could be explained by the fact that children initiating ART at their younger age are more likely to have an immature immune system, which causes rapid disease progression contrasted with a comparative group [22]. Additionally, HIV-status disclosure is the most important factor for positive treatment outcomes [23, 24]. However, in our study, HIV-status disclosure among children aged less than 10 years was very low. Furthermore, unlike younger children, older children might have the opportunity to get information about HIV/AIDS in school, enabling them to adhere to their treatment. On the other hand, in younger children, their drug has mainly been given by caregivers, and they could miss it.

However, this finding contradicts with researches done in Cameroon [14], and Tanzania [25] suggesting that older age at ART initiation was significantly associated with high virological failure. The possible explanation for the above noted differences could be due to the differences in inclusion criteria. For example, a study done in Cameroon included participants age lower than 18 years, whereas our study included children under 15 years.

This study also revealed opportunistic infections during follow-up were strongly associated with the occurrence of virological failure. This result is concordant with other reports from Tanzania [20] and Kenya [26], which demonstrated that co-morbidities such as TB (one of the opportunistic infections) were associated with the occurrence of virological failure. This is well-known that co-morbidities such as TB weaken the immune system among patients and rapidly increase HIV disease progression to its advanced stage. Additionally, although ART needs a lifelong commitment for its effectiveness, HIV-related co-morbidities could prohibit patients from getting their regular ART follow-up and treatment intake. There is an inverse linkage between viral replication and the efficiency of the immune system, resulting in acquiring virological failure [5, 27]. Finally, HIV-infected children with co-infections are at high risk for drug burden and drug-drug interactions which might lead to poor ART adherence and treatment failure, especially anti- TB- drugs [28].

History of ART treatment interruption/discontinuation was also significantly associated with the occurrence of virological failure. This is because ART suppresses the viral replication resulting in boosting the patient’s immunity, decreasing the incidence of OIs, and reducing the viral load [29]. Moreover, as ART helps boost HIV-infected individuals’ immunity, drug interruption could be associated with a decreased efficiency of the immune system and increased drug resistance risk. As a result, patients lose their ability to suppress viral replication and reduce viral load, finally leading to virological failure [30, 31]. In addition, this is due to the fact that interruption of antiretroviral therapy (ART), opportunistic infection, and WHO clinical stage are highly interrelated risk factors for treatment failure. As patients interrupt regular follow-up and discontinue taking drugs, their immune status becomes compromised. This implies, there is a high chance of acquiring opportunistic infections and increase the rate of viral replication as well, which leads to disease progress/advanced clinical stage and virological failure [29, 32].

This study also found that children classified as advanced WHO clinical staging (stage III and IV) were more likely to develop virological failure. This finding is in agreement with studies reported from Uganda [33] and Swaziland [18]. This might be due to the fact that children presenting with advanced WHO clinical stage at ART initiation are at higher risk of OIs, especially TB. The presence of OIs during ART initiation increase pill burden, which results in drug-drug interactions, and ultimately leads to treatment failure and early mortality. Studies suggested that HIV-infected individuals treated with anti-TB medications usually experience drug toxicity as compared to HIV-uninfected individuals [34, 35].

Finally, children who had fair/poor ART adherence level were also positively associated with virological failure. This finding is supported by studies conducted in Uganda [33], Tanzania [36], and Gabon [30]. If HIV-infected individuals failed to take their medication regularly and daily, they are at higher risk to developing drug resistance. This resulted in experience virological failure [29].

### Limitations

This study has some limitations that must be considered before interpreting results. Since the study was based on secondary data, some important variables (such as educational level and income) were missed. Thus, there might be information bias because of underreporting/missing data elements. There could also be selection bias during data collection as individuals with incomplete medical records were excluded. As the study used cross-sectional study design, it did not establish the possible cause and effect relationship between dependent and independent variables.

## Conclusion

This study demonstrated that, there was a high magnitude of virological failure among HIV positive children compared to the current Ethiopian ART guidelines target (not more than 10%). Children with poor/fair ART adherence level, history of ART treatment interruption, advanced baseline WHO clinical staging (stage III and IV), younger age, and opportunistic infections were found to be factors significantly associated with the occurrence of virological failure.

## Data Availability

The data sets used and/or analyzed during the current study are available from the Corresponding author on reasonable request.

## Abbreviations

AHC: Abay Health Center
AIDS: Acquired Immune Deficiency Syndrome
AOR: Adjusted odd ratio
APHI: Amhara Public Health Institute
ART: Antiretroviral Therapy
BHC: Bahir Dar Health Center
CI: Confidence Interval
COR: Crud odd ratio
CPT: Cotrimoxazole Preventive Therapy
EAC: Enhanced Adherence Counseling
FHCSH: Felege-Hiwot Comprehensive Specialized Hospital
HHC: Han Health Center
HIV: Human immunodeficiency Virus
IPT: Isoniazid Prophylaxis Therapy
IRB: Institutional Review Board
OI: Opportunistic Infection
SDGs: Sustainable Development Goals
SPSS: Statistical Package for Social science
TB: Tuberculosis
TF: Treatment Failure
UNAIDS: Joint United Nations Programme on HIV/ AIDS
VF: Virologic Failure
VLS: Viral Load Suppression
WHO: World Health Organization
